# Correction: Feasibility study of noninvasive assessment of emotional state in patients with ALS

**DOI:** 10.3389/fdgth.2026.1977072

**Published:** 2026-09-23

**Authors:** Marc Garbey, Quentin Lesport, Gülşen Öztosun, Megan Heidebrecht, Kamyab Pirouz, Elham Bayat

**Affiliations:** 1 Care Constitution Corp, Houston, TX, United States; 2 Laboratoire des Sciences de l'Ingénieur pour l'Environnement (LaSIE) UMR-CNRS 7356, University of La Rochelle, La Rochelle, France; 3 Department of Neurology & Rehabilitation Medicine, The George Washington University School of Medicine & Health Sciences, Washington, DC, United States; 4 The George Washington University School of Medicine & Health Sciences, Washington, DC, United States

**Keywords:** affective computing, amyotrophic lateral sclerosis, clinical trial, generative language, natural language processing, signal analysis, telemedicine

The title of this article was erroneously given as: Feasibility Study on a noninvasive assessment of ALS patient emotional state. The correct title of the article is Feasibility study of noninvasive assessment of emotional state in patients with ALS.

The patient exclusion criteria was erroneously omitted.

A correction has been made to the section **Methods**, *Human subjects*, Paragraph 1:

“Twenty-five subjects were recruited from the multidisciplinary ALS clinic at George Washington University (GWU). Eligibility required clinical and electrophysiological confirmation of Amyotrophic Lateral Sclerosis (ALS), age over 18 years, and the ability to provide written informed consent. Exclusion criteria included: an unstable medical condition that posed a risk to participation (per the examining physician's judgment), pre-diagnosis cognitive impairment, dementia, cardiovascular conditions related to arrythmias or a psychiatric illness. Patients with late-stage disease necessitating tube feeding, power wheelchair, or who had moderate to severe dysarthria were also excluded. All patients had to be able to talk in an intelligible way, so the disease is early stage with respect to that skill. The study protocol was approved by the GWU Institutional Review Board. The recruitment was done in two stages. The first cohort of 14 patients as described in our initial pilot study [9] that was used to get our first results on ALS patient management and a year later a second cohort to start the process of a longitudinal study published eventually later. This one-year interval between the two cohorts allows us to consider that the first and second visit of the three ALS patients involved in both cohorts will be considered as independent data points for the purpose of this study.”

There was a mistake in table 2 as published. In the last row the Patient Class was written as “Class 1#3” instead of “Class 1&3”. The corrected table 2 appears below.

The Generative AI statement was erroneously given as “The author(s) declared that generative AI was used in the creation of this manuscript. In this study, Generative Artificial Intelligence (AI) was employed for both methodological development and comparative analysis. Specifically, ChatGPT (OpenAI) was used to assist in the manual extraction and development of a key term dictionary for Natural Language Processing (NLP) topic classification. Additionally, current- generation large language models (LLMs), including ChatGPT, Gemini (Google), and Claude (Anthropic), were utilized to evaluate their ability to quantitatively score patient anxiety levels based on clinical transcripts. These LLM-generated scores were compared against established clinician ratings to assess the models’ reproducibility and reliability for clinical emotional assessment. The researchers confirm that while these tools assisted in specific data classification and analysis tasks, they did not replace expert clinical supervision or the primary data- driven findings of the study. Any alternative text (alt text) provided alongside figures in this article has been generated by Frontiers with the support of artificial intelligence and reasonable efforts have been made to ensure accuracy, including review by the authors wherever possible. If you identify any issues, please contact us.

The correct Generative AI statement is “The author(s) declared that generative AI was used in the creation of this manuscript. Large language models (LLMs) were used solely as an experimental tool in this study to compare LLM-generated scores with clinician-generated scores and to partially assist in generating the vocabulary dictionary used in the analysis. Generative AI tools were also used to support language editing, proofreading, and grammar correction of the manuscript. Generative AI was not used to generate, determine, or modify the study methodology, analyze or generate the study results, or draw scientific conclusions. All methodological decisions, data analysis, interpretation of results, and final manuscript content were reviewed and verified by the authors, who retain full responsibility for the accuracy and integrity of the work.

The Data Availability statement was erroneously given as “The raw data supporting the conclusions of this article will be made available by the authors, without undue reservation.”.

The correct Data Availability statement is “Due to privacy and confidentiality concerns, patient voice recordings and verbatim transcripts will not be provided, as these materials may contain direct or potentially identifiable patient information. To protect patient privacy while supporting transparency and reproducibility, we can provide an Excel file containing anonymized scores and relevant study data, with all direct patient identifiers removed. The anonymized dataset will be made available upon reasonable request to the corresponding author/s, subject to applicable ethical and institutional requirements.

The conflict of interest statement was erroneously given as “MG and QL were employed by Care Constitution Corp. The remaining author(s) declared that this work was conducted in the absence of any commercial or financial relationships that could be construed as a potential conflict of interest.” The correct Conflict of Interest statement is “MG and QL are employed by Care Constitution Corp. The remaining author(s) declared that this work was conducted in the absence of any commercial or financial relationships that could be construed as a potential conflict of interest.

The original version of this article has been updated.

**Table:** 

	Time in Clinic	Loudness	Sharpness	Mean Roughness	Median Roughness	Max Roughness	Mean Fluctuation	Median Fluctuation	Max Fluctuation
Class 1&2	0.03 left	0.08 right	0,33 right	0.21 left	0.5 left	0.19 left	0.36 left	0.33 left	0.41 left
Class 2&3	0.2 left	0.05 right	0.27 right	0.03 left	0.07 left	0.02 left	0.09 left	0.08 left	0.09 left
Class 1&3	0.016 left	0.016 right	0.09 right	0.06 left	0.09 left	0.03 left	0.09 left	0.09 left	0.14 left

