# Supplementary material for: Feasibility study of noninvasive assessment of emotional state in patients with ALS
Source: Front Digit Health. 2026 Aug 14;8:1886561. doi: 10.3389/fdgth.2026.1886561 (PMC13522213; doi:10.3389/fdgth.2026.1886561)
Supplement: Supplementary file 1 [file Datasheet1.docx]

Supplementary Material

# 1 Supplementary Data

**LLM Prompt with DATA SET 0**

From the following conversation, can you score successively from 0 to 3 the anxiety of the patient related to 1. breathing and Mobility issues, 2 Nutrition issues, 3 Finance issues. 0 meaning no anxiety, 1 meaning mild anxiety, 2 meaning moderate anxiety and 3 meaning extreme anxiety:

**LLM Prompt with DATA SET 1**

From the following conversation, can you score successively from 0 to 3 the anxiety of the patient related to 1. breathing issues, 2 Mobility issues, 3 Nutrition issues, 4 Finance issues. 0 meaning no anxiety, 1 meaning mild anxiety, 2 meaning moderate anxiety and 3 meaning extreme anxiety

# 2 Supplementary Figures and Tables

**Supplementary Figure 1.** The figure legends are required to have the same font as the main text, 12 point normal Times New Roman, single spaced. Please use a single paragraph for each legend and prepare the figures keeping in mind the PDF layout.

**Supplementary Figure** **2**: Comparison of the Distribution of the 4 Question Scores between Clinician (top left) and LLM with respectively Chat GPT (top right), Gemini (bottom left) and Claude (bottom right). We observe a larger distribution of LLM score than for the clinical one: LLM score are usually higher than clinical one, Claude being the closest to the clinical score.
